# Arg462Gln and Asp541Glu polymorphisms in *ribonuclease L* and prostate cancer risk: a meta-analysis

**DOI:** 10.1016/S1674-8301(10)60049-8

**Published:** 2010-09

**Authors:** Yuanyuan Mi, Qianqian Yu, Zhichao Min, Bin Xu, Lifeng Zhang, Wei Zhang, Ninghan Feng, Lixin Hua

**Affiliations:** a Department of Urology, the First Affiliated Hospital, Nanjing Medical University, Nanjing 210029, Jiangsu Province, China; b Department of Ophthalmology, Wuxi People's Hospital Affiliated with Nanjing Medical University, Wuxi 214023, Jiangsu Province, China

**Keywords:** ribonuclease L, polymorphism, prostate cancer, risk, meta-analysis

## Abstract

**Objective:**

The association between *ribonuclease L (RNASEL)* gene polymorphisms and prostate cancer risk has been widely reported, but the results of these studies remained controversial and underpowered. We performed a meta-analysis of 28 studies to evaluate the association between Arg462Gln and Asp541Glu polymorphisms in the *RNASEL* gene and prostate cancer risk.

**Methods:**

Odds ratios (ORs) with 95% confidence intervals (CIs) were estimated to assess the association between RNASEL polymorphisms and prostate cancer risk.

**Results:**

A significantly increased prostate cancer risk was found for the Arg462Gln polymorphism in Africans (Gln/Gln *vs* Arg/Arg: OR = 2.50, 95%CI = 1.28-4.87; Gln/Gln *vs* Gln/Arg + Arg/Arg: OR = 2.54, 95%CI = 1.30-4.95), but not in Europeans and Asians. Additionally, the Asp541Glu polymorphism was associated with increased total prostate cancer risk (Glu-allele *vs* Asp-allele: OR = 1.04, 95%CI = 1.01-1.07; Glu/Glu *vs* Asp/Asp: OR = 1.22, 95%CI = 1.03-1.46; Glu/Glu *vs* Glu/Asp + Asp/Asp: OR = 1.09, 95%CI = 1.02-1.16). In the stratified analysis for the Asp541Glu polymorphism, there was a significantly increased prostate cancer risk in Africans and Europeans, and in hospital-based prostate cancer cases.

**Conclusion:**

The meta-analysis results showed evidence that *RNASEL* Arg462Gln and Asp541Glu polymorphisms are associated with prostate cancer risk and could be low-penetrance prostate cancer susceptibility biomarkers.

## INTRODUCTION

Prostate cancer is the most common non-skin cancer and the second leading cause of cancer-related-death of men in the USA, with an estimated 192,000 new cases and 27,000 deaths annually[1]. The underlying etiology of prostate cancer remains poorly understood, with both genetic predisposition and environmental factors likely to play a role. A recent study showed that prostate cancer patients with good and poor survival tend to aggregate in families, providing evidence of heritability in the prognosis of prostate cancer[2]. Despite this strong evidence for a genetic component in prostate cancer, little progress has been made in identifying a major gene or genes[3].

The *ribonuclease L* (*RNASEL*) gene locates on chromosome 1q24-25 and is comprised of 741 amino acids. One of the candidate genes within the hereditary prostate cancer gene region is the gene encoding 2′-5′-oligoadenylate-dependent RNASEL (MIM 601518 and 180435)[4]–[7], a constitutively expressed latent endonuclease that is involved in the antiviral and pro-apoptotic activities of the interferon-inducible 2-5A system, cellular viral defense, single-stranded RNA cleavage and tumor suppressor activities as stress could cause apoptosis, cell proliferation and regulation of protein synthesis[8]–[13].

There are different polymorphism sites in the *RNASEL* gene. Sequence analysis of *RNASEL* identified two common missense mutations: Arg462Gln (rs486907) and Asp541Glu (rs627928). The Arg462Gln polymorphism reduces the ability of the cell to cause apoptosis in response to activation by 2-5 (A) and the enzymatic activity is also three times less than normal[14], whereas the Asp541Glu variant has no known effects on RNASEL function[15]. Previously, Li *et al.*[16] performed a meta-analysis of the association of these two polymorphisms of *RNASEL* and prostate cancer risk, and found that the Asp541Glu polymorphism was associated with an increased risk of prostate cancer in Caucasians. However, they failed to find a significant association between the Arg462Gln polymorphism and prostate cancer risk in Africans. The main reason to explain this conclusion may be that they did not include any case-control studies with the African population.

Hitherto, there have been 15 studies that investigated the Arg462Gln polymorphism[17]–[27],[29], and 13 studies that investigated the Asp541Glu polymorphism and the risk of prostate cancer[17]–[20],[23]–[29]. Taking into consideration the extensive evidence for a role of RNASEL in prostate cancer, we performed a meta-analysis of all eligible case-control studies in order to derive a more precise estimation of the association of the Arg462Gln and Asp541Glu polymorphisms in RNASEL and prostate cancer risk.

## MATERIALS AND METHODS

### Publication search

We attempted to include all the case-control studies published to date on the association between *RNASEL* gene polymorphisms and prostate cancer risk. Eligible studies were identified by searching the electronic literature in PubMed for relevant reports (the last search update was 7 May 2010, using the search terms “*RNASEL*”, “polymorphism” and “prostate cancer”). A total of 64 articles were retrieved, of which 13 articles met the inclusion criteria for studies examining the association between the *RNASEL* Arg462Gln and/or Asp541Glu polymorphism and prostate cancer risk.

### Inclusion and exclusion criteria

The following inclusion criteria were used for literature selection: 1) studies evaluating the *RNASEL* Arg462Gln and/or Asp541Glu polymorphism and prostate cancer risk; 2) case-control studies; 3) studies that contained information about available genotype frequency that could help evaluate the results in the papers; 4) studies that were published in English; 5) studies with full-text manuscripts. Major exclusion criteria were: 1) studies with no control population; 2) studies with no available data on genotype frequency; 3) studies with a clear bias of accrual.

### Data extraction

Data was extracted from each study by two investigators independently according to the prespecified inclusion criteria. Data extracted from these articles included: first author, year of publication, origin, ethnicity, source of cases (population-based and hospital-based), sample size (case/control), frequency of genotype in cases and controls, and Hardy–Weinberg equilibrium of controls. Different ethnic descents were categorized as European, Asian and African.

### Statistical analysis

Odds ratios (ORs) with 95% confidence intervals (CIs) were used to measure the strength of the association between the *RNASEL* Arg462Gln or Asp541Glu polymorphism and prostate cancer risk based on the genotype frequencies in cases and controls. We explored the association between allele Gln of *RNASEL* Arg462Gln or allele Glu of Asp541Glu and prostate cancer risk, as well as the heterozygote comparison Gln/Arg *vs* Arg/Arg or Glu/Asp *vs* Asp/Asp, the homozygote comparison Gln/Gln *vs* Arg/Arg or Glu/Glu *vs* Asp/Asp and the risk of (Gln/Gln+Gln/Arg) *vs* Arg/Arg or (Glu/Glu + Glu/Asp) *vs* Asp/Asp and Gln/Gln *vs* (Gln/Arg +Arg/Arg) or Glu/Glu *vs* (Glu/Asp + Asp/Asp) in dominant and recessive models, respectively. Subgroup analysis was performed by ethnicity and source of cases (population-based and hospital-based) for the Arg462Gln or Asp541Glu polymorphism.

The fixed effects model and the random effects model were used to calculate the pooled OR. Heterogeneity assumption was evaluated with a chi-square-based *q* test among the studies. A *P* value of more than 0.05 for the *q*-test indicated a lack of heterogeneity among the studies. A random effects model (DerSimonian and Laird method[30]) was used when the *P* value for the heterogeneity test was <0.05; otherwise, a fixed effects model (Mantel-Haenszel method[31]) was used. The departure of frequencies of *RNASEL* polymorphisms from expectation under Hardy-Weinberg equilibrium was assessed by χ^2^ test in controls, and *P* < 0.05 was considered statistically significant. Publication bias was assessed with the Egger test[32], and *P* < 0.05 was considered statistically significant. All statistical tests for this meta-analysis were performed with the Stata software (Version 10.0, StataCorp LP, USA).

## RESULTS

### Eligible studies

Overall, 13 articles (28 studies, 13 different first authors) were identified for evaluating the association of the *RNASEL* Arg462Gln or Asp541Glu polymorphism with risk for prostate cancer. The characteristics of the studies of the Arg462Gln or Asp541Glu polymorphism are summarized in ***Table 1*** **and *2*** , respectively. According to the inclusion criteria, there were no genotypes of cases and controls in Wang *et al.*[26], but they were provided in the meta-analysis of Li *et al.*[16], so we included this case-control study in our meta-analysis. Breyer *et al.*[33] failed to provide the distribution of alleles. Therefore, there are 15 eligible studies with 7,461 cases and 6,963 controls concerning the Arg462Gln polymorphism and 13 studies with 5,353 cases and 4,321 controls concerning the Asp541Glu polymorphism. For the Arg462Gln polymorphism, there were 10 studies of European population[17],[19],[20],[22]–[24],[26],[27], 4 studies of African population[18],[19],[21],[29] and one study of Asian population[25]. Hospital-based cases were used in 7 studies. For the Asp541Glu polymorphism, there were nine studies of European population[17],[19],[20],[23],[24],[26]–[28], 3 studies of African population[18],[19],[29] and one study of Asian population[25]. Hospital-based cases were used in 8 studies. With the exception of one study[17], the distribution of genotypes in the controls was consistent with the Hardy-Weinberg equilibrium.

**Table 1:** Charateristics of studies of the *RNASEL* Arg462Gln polymorphism included in this meta-analysis

First author	Year	Origin	Ethnicity	Source of cases	Sample size (case/control)	Cases			Controls			*P*^b^
						QQ	QR	RR	QQ	QR	RR	
Beuten[17]	2010	USA	European	HB	156/224	17	64	75	7	91	126	0.048
Robbins[18]	2008	USA	African	HB	243/296	5	55	183	5	66	225	0.950
Shook[19]	2007	USA	European	HB	430/503	60	183	187	57	225	221	0.981
Shook[19]	2007	USA	European	HB	150/239	16	62	72	7	96	136	0.093
Shook[19]	2007	USA	African	HB	68/145	10	13	45	3	31	111	0.633
Cybulski[20]	2006	Canada	European	PB	737/511	116	376	245	82	252	177	0.625
Daugherty[21]	2007	USA	European	PB	1,116/1,344	148	505	463	188	602	554	0.235
Daugherty[21]	2007	USA	African	PB	98/380	2	23	73	5	98	277	0.261
Nam[22]	2005	Canada	European	PB	996/1,092	110	409	477	112	459	521	0.464
Maier^a[23]^	2005	Germany	European	HB	363/207	59	171	133	37	97	73	0.629
Wiklund^a[24]^	2004	Sweden	European	PB	1,622/796	247	778	597	115	384	297	0.611
Nakazato^a[25]^	2003	Japan	Asian	PB	101/105	0	32	69	8	26	71	0.085
Wang^a[26]^	2002	USA	European	HB	918/493	102	427	389	67	233	193	0.802
Rokman^a[27]^	2002	USA	European	PB	233/176	39	106	88	23	84	69	0.745
Shea[29]	2008	USA	African	PB	230/452	2	41	187	2	88	362	0.168

^a^ Studies were reported by Li *et al*; ^b^ Hardy-Weinberg equilibrium of controls.

RNASEL: Ribonuclease L; HB: Hospital-based; PB: Population-based; QQ: Gln/Gln; QR: Gln/Arg ; RR: Arg/Arg.

**Table 2:** Characteristics of studies of the *RNASEL* Asp541Glu polymorphism included in this meta-analysis

First author	Year	Origin	Ethnicity	Source of cases	Sample size (case/control)	Cases			Controls			*P*^b^
						EE	ED	DD	EE	ED	DD	
Beuten[17]	2010	USA	European	HB	156/227	45	70	41	48	120	59	0.368
Robbins[18]	2008	USA	African	HB	243/296	38	102	103	24	129	143	0.495
Shook[19]	2007	USA	European	HB	430/484	140	190	100	139	254	91	0.187
Shook[19]	2007	USA	European	HB	150/242	43	66	41	48	125	69	0.525
Shook[19]	2007	USA	African	HB	68/146	9	28	31	15	60	71	0.661
Cybulski[20]	2006	Canada	European	PB	737/511	254	372	111	168	259	84	0.344
Rokman^a[27]^	2002	USA	European	PB	233/176	78	126	29	56	91	29	0.434
Wang^a[26]^	2002	USA	European	HB	929/508	181	476	272	107	228	173	0.050
Wiklund^a[24]^	2004	Sweden	European	PB	1,563/791	522	768	273	257	372	162	0.199
Maier^a[23]^	2005	Germany	European	HB	363/207	125	176	62	69	97	41	0.514
Nakazato^a[25]^	2003	Japan	Asian	PB	101/105	51	32	18	59	43	3	0.138
Noonan-Wheeler ^a[28]^	2006	USA	European	HB	150/170	55	73	22	44	93	33	0.198
Shea [29]	2008	USA	African	PB	230/458	26	97	107	40	201	217	0.496

^a^ Studies were reported by Li *et al*; ^b^ Hardy-Weinberg equilibrium of controls.

RNASEL: ribonuclease L; HB: Hospital-based; PB: Population-based; EE: Glu/Glu; ED: Glu/Asp; DD: Asp/Asp.

### The *RNASEL* Arg462Gln polymorphism

**Fig 1:**
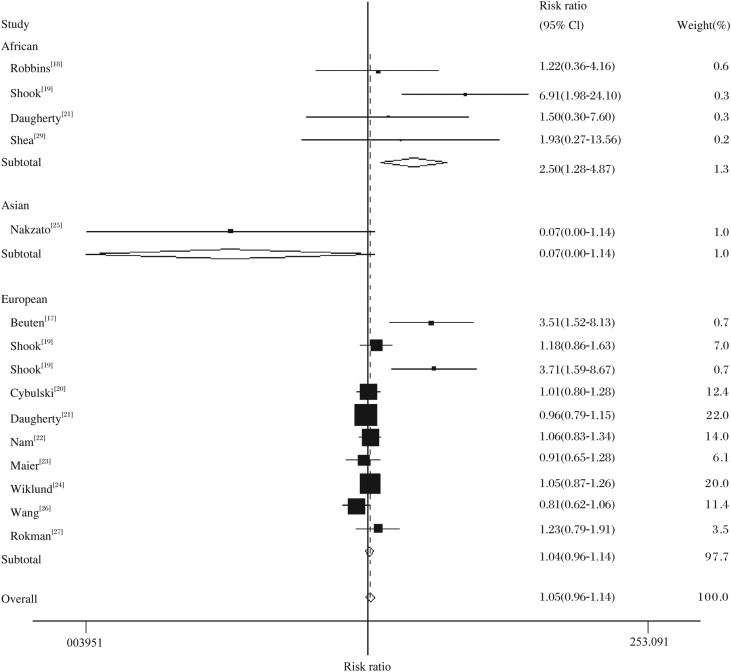
Forest plot of prostate cancer risk associated with the *RNASEL* Arg462Gln polymorphism (Gln/Gln *vs* Arg/Arg) stratified by ethnicity. The squares and horizontal lines correspond to the study-specific OR and 95% CI. The area of the squares reflects the weight (inverse of the variance). The diamond represents the summary OR and 95% CI.

In the overall analysis, no association could be observed between prostate cancer risk and the variant genotype of *RNASEL* Arg462Gln in different genetic models: in the comparison of the Gln allele *vs* the Arg allele (random-effects OR = 1.05, 95% CI = 0.97-1.13, *P*_heterogeneity_ = 0.028), the heterozygote comparison (Fixed-effects OR = 1.00, 95% CI = 0.96-1.04, *P*_heterogeneity_ = 0.997), the homozygote comparison (Random-effects OR = 1.20, 95% CI = 0.96-1.50, *P*_heterogeneity_ = 0.001), or the dominant models (fixed-effects OR = 1.01, 95% CI = 0.98-1.04, *P*_heterogeneity_ = 0.740), or the recessive models (Random-effects OR = 1.18, 95% CI = 0.96-1.46, *P*_heterogeneity_ = 0.001). However, a significant association between the Arg462Gln polymorphism and prostate cancer in African population was found in the stratified analysis by ethnicity, the homozygote comparison (Fixed-effects OR = 2.50, 95% CI = 1.28-4.87, *P* = 0.231 for heterogeneity; see ***Table 3*** **and** ***Fig. 1***) and the recessive models (Fixed-effects OR = 2.54, 95% CI = 1.30-4.95, *P*_heterogeneity_ = 0.224). In the subgroup analysis of the source of cases, association was found in hospital-based cases: the homozygote comparison (Random-effects OR = 1.74, 95% CI = 1.00-3.03, *P*_heterogeneity_ = 0.000) and the recessive models (Random-effects OR = 1.69, 95% CI = 1.01-2.83, *P*_heterogeneity_ = 0.000).

**Table 3:** Stratified analyses of the *RNASEL* Arg462Gln polymorphism and prostate cancer risk

Variables	*n^a^*	Cases/Controls	Q- *vs* R-allele		QQ *vs* RR		QR *vs* RR		QQ *vs* QR+RR		QQ+QR *vs* RR	
			OR(95%CI)	*P^b^*	OR(95%CI)	*P^b^*	OR(95%CI)	*P^b^*	OR(95%CI)	*P^b^*	OR(95%CI)	*P^b^*
Total	15	7461/6963	1.05(0.97-1.13)	0.028	1.20(0.96-1.50)	0.001	1.00(0.96-1.04)	0.997^c^	1.18(0.96-1.46)	0.001	1.01(0.98-1.04)	0.740^c^
Ethnicity												
African	4	639/1273	1.10(0.92-1.32)	0.056^c^	2.50(1.28-4.87)	0.231 ^c^	0.96(0.80-1.16)	0.959^c^	2.54(1.30-4.95)	0.224^c^	1.03(0.86-1.22)	0.433^c^
Asian	1	101/105	0.75(0.45-1.25)	-	0.06(0.00-1.07)	-	1.27(0.69-2.34)	-	0.06(0.00-0.99)	-	0.97(0.54-1.74)	-
European	10	6721/5585	1.01(0.98-1.05)	0.061^c^	1.13(0.92-1.38)	0.006	1.00(0.96-1.04)	0.978^c^	1.11(0.93-1.34)	0.009	1.01(0.98-1.04)	0.594^c^
Source of cases												
Population-based	8	5133/4856	1.00(0.96-1.04)	0.948^c^	1.02(0.92-1.12)	0.597^c^	1.00(0.96-1.04)	0.985^c^	1.01(0.91-1.13)	0.511^c^	1.00(0.97-1.04)	0.999^c^
Hospital-based	7	2328/2107	1.18(0.97-1.44)	0.001	1.74(1.00-3.03)	0.000	1.00(0.93-1.07)	0.902^c^	1.69(1.01-2.83)	0.000	1.02(0.96-1.08)	0.134^c^

^a^ Number of comparisons; ^b^ *P* value of *q*-test for heterogeneity test; ^c^ Random effects model was used when *P* for the heterogeneity test was <0.05; otherwise, fixed effects model was used.

RNASEL: ribonuclease L; 95% CI: 95% confidence interval; OR: odds ratio; QQ: Gln/Gln; QR: Gln/Arg; RR: Arg/Arg.

### Test of heterogeneity of the *RNASEL* Arg462Gln polymorphism

There was significant heterogeneity for the Gln allele *vs* the Arg allele (*P*_heterogeneity_ = 0.028) in the homozygote comparison (*P*_heterogeneity_ =0.001) and the recessive models (*P*_heterogeneity_ = 0.001), but not in the heterozygote comparison (*P*_heterogeneity_ = 0.997) and the dominant models (*P*_heterogeneity_ = 0.740).

### The *RNASEL* Asp541Glu polymorphism

**Fig 2:**
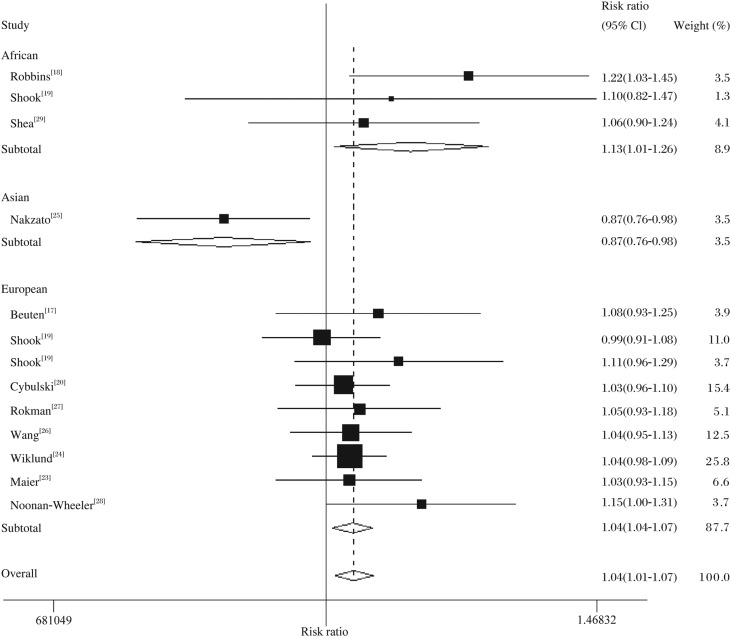
Forest plot of prostate cancer risk associated with the *RNASEL* Asp541Glu polymorphism (the Glu-allele *vs* the Asp-allele) stratified by ethnicity. The squares and horizontal lines correspond to the study-specific OR and 95% CI. The area of the squares reflects the weight (inverse of the variance). The diamond represents the summary OR and 95% CI.

In the overall analysis, the 541Glu allele was associated with increased prostate cancer risk, as compared with the Asp541 allele (Fixed-effects OR = 1.04, 95% CI = 1.01-1.07, *P*_heterogeneity_ = 0.164; ****Table 4**** **and** ***Fig. 2***), as well as in the homozygote comparison (random-effects OR = 1.22, 95% CI = 1.03-1.46, *P*_heterogeneity_ = 0.004) and the recessive models (Fixed-effects OR = 1.09, 95% CI = 1.02-1.16, *P*_heterogeneity_ = 0.104), but not in the heterozygote comparison (Random-effects OR = 1.03, 95% CI = 0.88-1.21, *P*_heterogeneity_ = 0.018) and the dominant models (Random-effects OR = 1.10, 95% CI = 0.96-1.26, *P*_heterogeneity_ = 0.016). Specifically, there was a significantly increased risk between prostate cancer and the *RNASEL* Asp541Glu polymorphism in ethnicity and source of cases, *e.g*. in the comparison of the Glu allele *vs* the Asp allele in African population (Fixed-effects OR = 1.13, 95% CI = 1.01-1.26, *P*_heterogeneity_ = 0.458), European population (Fixed-effects OR = 1.04, 95% CI = 1.01-1.07, *P*_heterogeneity_ = 0.828) and among studies with hospital-based prostate cancer cases (Fixed-effects OR = 1.06, 95% CI = 1.02-1.11, *P*_heterogeneity_ = 0.401). The same results were also observed in the homozygote comparison and the recessive models. Furthermore, there was significant association between the Asp541Glu polymorphism and hospital-based prostate cancer in the homozygote comparison and recessive models (data not shown).

**Table 4:** Stratified analyses of the *RNASEL* Asp541Glu polymorphism and prostate cancer risk

Variables	*n^a^*	Cases/Controls	E- *vs* D-allele		EE *vs* DD		ED *vs* DD			EE *vs* ED+DD		EE+ED *vs* DD	
			OR(95%CI)	*P^b^*	OR(95%CI)	*P^b^*	OR(95%CI)		*P^b^*	OR(95%CI)	*P^b^*	OR(95%CI)	*P^b^*
Total	13	5353/4321	1.04(1.01-1.07)	0.164^c^	1.22(1.03-1.46)	0.004	1.03(0.88-1.21)		0.018	1.09(1.02-1.16)	0.104^c^	1.10(0.96-1.26)	0.016
Ethnicity													
African	3	541/900	1.13(1.01-1.26)	0.458^c^	1.49(1.11-2.00)	0.433^c^	1.02(0.90-1.15)		0.897^c^	1.53(1.13-2.08)	0.457 ^c^	1.06(0.96-1.17)	0.699 ^c^
Asian	1	101/105	0.60(0.39-0.92)	-	0.14(0.04-0.52)	-	0.12(0.03-0.46)		-	0.80(0.46-1.38)		0.14(0.04-0.48)	-
European	9	4711/3316	1.04(1.01-1.07)	0.828 ^c^	1.07(1.01-1.13)	0.742^c^	1.03(0.99-1.07)		0.113^c^	1.07(1.00-1.15)	0.246^c^	1.03(1.00-1.05)	0.396 ^c^
Source of cases													
Population-based	5	2864/2041	1.02(0.99-1.06)	0.106 ^c^	1.09(0.76-1.54)	0.027^c^	1.02(0.74-1.41)		0.013	1.04(0.95-1.13)	0.718^c^	1.05(0.77-1.42)	0.015
Hospital-based	8	2489/2280	1.06(1.02-1.11)	0.401^c^	1.11(1.02-1.21)	0.150^c^	1.02(0.96-1.07)		0.125^c^	1.16(1.05-1.28)	0.065^c^	1.03(0.99-1.07)	0.253^c^

^a^ Number of comparisons; ^b^ *P* value of *q*-test for heterogeneity test; ^c^ Random effects model was used when *P* for the heterogeneity test was <0.05; otherwise, fixed effects model was used.

RNASEL: ribonuclease L; 95% CI: 95% confidence interval; OR: odds ratio; E: Glu; D: Asp; EE: Glu/Glu; ED: Glu/Asp; DD: Asp/Asp.

### Test of heterogeneity of the *RNASEL* Asp541Glu polymorphism

There was significant heterogeneity in the homozygote comparison (*P*_heterogeneity_ = 0.004), the heterozygote comparison (*P*_heterogeneity_ = 0.018) and the dominant models (*P*_heterogeneity_ = 0.016), but not in the Glu-allele *vs* the Asp-allele (*P*_heterogeneity_ = 0.164) and the recessive models (*P*_heterogeneity_ = 0.104).

### Sensitivity analysis

Sensitivity analysis was used to determine whether modification of the inclusion criteria of the meta-analysis affected the final results. These were carried out by limiting the meta-analysis to the studies conforming to Hardy-Weinburg equilibrium and altering corresponding statistical variables and analysis models. Moreover, no other single study influenced the summary OR qualitatively as indicated by sensitivity analysis.

### Bias diagnosis

**Fig 3:**
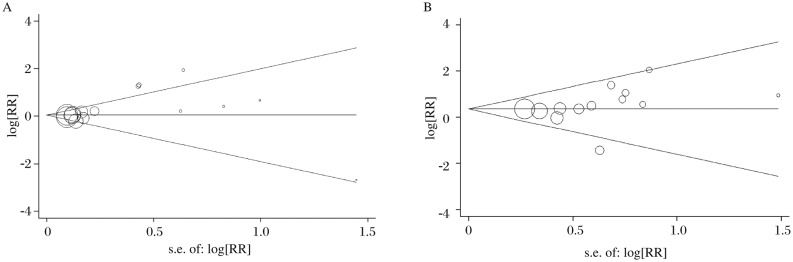
Funnel plot analysis for publication bias. Each point represents a separate study for the indicated association. A: a funnel plot for allele contrast (Gln/Gln *vs* Arg/Arg) of the Arg462Gln polymorphism in overall analysis. B: a funnel plot for allele contrast (the Glu-allele *vs* the Asp-allele) of the Asp541Glu polymorphism in overall analysis.

The Begg's funnel plot and Egger's test were performed to assess the publication bias of the literature. The shape of the funnel plots did not reveal any evidence of obvious asymmetry in the homozygote comparison of the Arg462Gln and the Glu-allele *vs* the Asp-allele of Asp541Glu. Then, Egger's test was used to provide statistical evidence of funnel plot symmetry. The results still did not reveal any evidence of publication bias (Gln/Gln *vs* Arg/Arg, *t* = 1.91, *P* = 0.079; the Glu-allele *vs* the Asp-allele, *t* = 1.08, *P* = 0.305. ***Fig. 3***).

## DISCUSSION

*RNASEL* encodes the RNASEL protein, a constitutively expressed latent endoribonclease that mediates the IFN-inducible 2–5A system. The protein functions partly as a tumor suppressor, acting to induce apoptosis of cancer cells, is associated with the activity of interferons. Casey *et al.*[14] and Xiang *et al.*[15] found that deficiency in the Arg462Gln variant is correlated with a decrease in enzyme dimerization to the active form and a reduction in the ability to cause apoptosis. However, the Asp541Glu variant has no effect on RNASEL function. The rapid growth of *RNASEL* genetic studies provides numerous opportunities for studiny disease association. The present meta-analysis involved 7,461 cases and 6,963 controls concerning the Arg462Gln polymorphism in the protein kinase region of RNASEL, and 5,353 cases and 4,321 controls concerning the Asp541Glu polymorphism in the ribonuclease domain of RNASEL. We then explored the association between these two potentially functional polymorphisms of *RNASEL* and prostate cancer risk.

Previously, Li *et al.*[16] performed a meta-analysis of the association of these two variants in *RNASEL* and prostate cancer risk, which included 11 case-control studies (each study contains the frequency of genotypes), and suggested that the Asp541Glu polymorphism was associated with an increased risk of prostate cancer in Caucasians. In this article, the authors divided prostate cancer into familial/hereditary prostate cancer and familial/sporadic prostate cancer. In our meta-analysis, novel case-control studies in the last 4 y were included, and the familial/hereditary and familial/sporadic prostate cancer subjects were used together. The only Asian study[29] found decreased familial prostate cancer risk in Japanese with the genotype Gln/Gln. Larger studies involving a wider spectrum of Asian populations are needed for a more definitive evaluation of the relationship between the Arg462Gln polymorphism and prostate cancer risk in Asians.

The true role of the *RNASEL* Arg462Gln and Asp541Glu polymorphism and their influence on prostate cancer risk are similarly controversial. Genotype frequency of various polymorphic loci may exhibit racial differences. For our purposes, we divided the world's population into three broad racial groups, Asian, African and European[33].

The *RNASEL* Arg462Gln polymorphism was implicated in up to 13% of prostate cancer cases, with the resultant enzyme 3 times less active than the wildtype enzyme, and an association was found among Europeans and Africans between the *RNASEL* Arg462Gln polymorphism and sporadic prostate cancer risk[14]. Our results suggested that the *RNASEL* Arg462Gln polymorphism was associated with prostate cancer risk in the African population rather than in European and Asian population, which confirmed the hypothesis described above. Moreover, our analysis suggested that the Asp541Glu polymorphism was associated with increased prostate cancer risk among Europeans and Africans but not among Asians in all genetic models, although Asp541Glu produced similar levels of RNASEL activity to those of the wildtype enzyme[15]. It may be a reflection of the differences in genetic backgrounds and gene-environment interactions in the etiology. Other factors such as time-lag bias and publication bias may also have played a role. In time-lag bias, studies with ‘negative’ results take longer time to be published, whereas studies with ‘positive’ results are published much more quickly[34]. In publication bias, small studies with ‘negative’ results are never published, whereas equally small studies with similar quality but ‘positive’ results would appear in the literature[35]–[36]. We examined these possibilities and found that ‘positive’ studies were reported more in 462Gln and 541Glu allele carriers' studies (especially in the European and African studies).

In the source of cases, interestingly, we also found evidence for the association between the Arg462Gln polymorphism and prostate cancer risk using hospital-based cases, the same as the Asp541Glu polymorphism.

Some limitations of this meta-analysis should be mentioned. First of all, the number of published studies included in our meta-analysis was not sufficiently large for a comprehensive analysis, particularly for the Asian population. Further studies should be carried out to confirm such an effect in Asians. Second, pub-lication bias might have occurred, and our Egger's test results may have a substantial risk of being affected by such a bias. Third, the interactions between gene–gene, gene-environment and even different polymorphic loci of the same gene may modulate prostate cancer risk. Fourth, in some *RNASEL* polymorphism studies, a small number of cases and controls were included. Fifth, in some original studies, the authors divided the prostate cancer into two groups: familial/hereditary prostate and familial/sporadic prostate cancer, whereas, in our meta-analysis, we grouped them together, which may have influenced the results. Sixth, our meta-analysis was based on unadjusted estimates. A more precise analysis should be conducted if individual information, including other covariates, such as age, gender and prostate cancer stage, becomes available. In spite of these issues, our meta-analysis also had two advantages. First, substantial numbers of cases and controls were pooled from different studies, which significantly increased the statistical power of the analysis. Second, the quality of case–control studies included in the current meta-analysis was satisfactory based on our selection criteria.

In summary, the present meta-analysis found novel evidence that the *RNASEL* Arg462Gln polymorphism is associated with increased prostate cancer risk in Africans; moreover, the *RNASEL* Asp541Glu polymorphism is associated with prostate cancer risk in Europeans and Africans, confirming the results of previous meta-analyses. These two polymorphisms may be low-penetrance susceptibility biomarkers for prostate cancer. Further prospective studies with a larger population of participants worldwide are expected to examine associations between these two polymorphisms in *RNASEL* and prostate cancer.
